# First isolation of verocytotoxin-producing *Escherichia coli* O157:H7 from sports animals in Southern Thailand

**DOI:** 10.14202/vetworld.2022.2275-2284

**Published:** 2022-09-23

**Authors:** Jirarat Songsri, Wanida Mala, Sueptrakool Wisessombat, Kesinee Siritham, Sahida Cheha, Nattita Noisa, Tuempong Wongtawan, Wiyada Kwanhian Klangbud

**Affiliations:** 1 Department of Medical Technology, School of Allied Health Sciences, Walailak University, Nakhon Si Thammarat, 80160 Thailand; 2 Center of Excellence Research for Melioidosis and Microorganisms, Walailak University, Nakhon Si Thammarat, 80160 Thailand; 3 Department of Veterinary Medicine, Akkhraratchakumari Veterinary College, Walailak University, Nakhon Si Thammarat, 80160 Thailand

**Keywords:** *Escherichia coli* O157:H7, fighting bulls, fighting cocks, riding horses, sport animal, verocytotoxin

## Abstract

**Background and Aim:**

*Escherichia coli* O157:H7 is enterohemorrhagic *E. coli*, which produces verocytotoxin or Shiga toxin. It is a well-known cause of severe diseases in humans worldwide. Cattle and other ruminants are the main reservoirs of this organism. Sports animals, such as fighting bulls, riding horses, and fighting cocks, are economic animals in Southern Thailand. This study aimed to identify *E. coli* O157:H7 from the rectal swabs of these sports animals and determine the antimicrobial susceptibility patterns of isolated bacteria.

**Materials and Methods:**

The rectal swabs were collected from 34 fighting bulls, 32 riding horses, and 31 fighting cocks. The swabs were cultured on MacConkey (MAC) Agar; the suspected colonies were then identified by VITEK^®^ 2 GN card, and the antimicrobial susceptibility was tested by VITEK^®^ 2 AST N194 in VITEK^®^ 2 Compact automation. *Escherichia coli* O157:H7 was confirmed by culturing on sorbitol MAC agar, the ability to grow at 44°C, and the presence of H7 antigen. In addition, the *eaeA* (*E. coli* attaching and effacing), along with *stx1* and *stx2* (Shiga cytotoxins) genes, were determined using polymerase chain reaction. Finally, the cytotoxicity of Shiga toxin was confirmed using the Vero cytotoxicity test.

**Results:**

Fifty-five suspected isolates (56.70%), which were collected from 19 fighting bulls (55.88%), 13 riding horses (40.63%), and 23 fighting cocks (71.13%), were identified as *E. coli*. However, one sample (Bull H9/1) from fighting bulls had an equal confidence level (50%) for *E. coli* and *E. coli* O157. The confirmation of this isolate demonstrated that it was sorbitol non-fermenter, could assimilate L-lactate, was unable to grow well at 44°C, and reacted with anti-serum to H7 antigen. In addition, it was positive with *stx2* and *eaeA* genes, and the toxin affected Vero cells by a dose-dependent response. The antimicrobial susceptibility test revealed that five out of 55 (9.09%) *E. coli* isolates were resistant to antimicrobial agents. All five isolates (21.74%) were collected from fighting cocks. *Escherichia coli* Cock H4/3 was only one of the five isolates resistant to three antimicrobial agents (ciprofloxacin, moxifloxacin, and trimethoprim/sulfamethoxazole). Fortunately, it was not multidrug-resistant bacteria.

**Conclusion:**

This is the first report on detection of *E. coli* O157:H7 in fighting bulls and antibiotic-resistant characteristic of *E. coli* in fighting cocks in Southern Thailand. This research is beneficial in preventing the dissemination of *E. coli* O157:H7 or antimicrobial agent-resistant *E. coli* in sports animals and humans.

## Introduction

*Escherichia coli* is a facultative anaerobic gram-negative bacillus and a member of the family Enterobacteriaceae. *Escherichia coli* is classified as a serotype based on the type of somatic (O) and flagella (H) antigens. Approximately 186 and 53 types of O and H antigens, respectively, have been reported so far. This bacterium is mostly a normal flora found in the environment, foods, and the gastrointestinal tracts of humans and animals. However, some serotypes cause diarrhea with mild-to-cholera-like symptoms, gastroenteritis, urinary tract infections, respiratory tract infection, neonatal meningitis, and other illnesses. The diarrheagenic *E. coli* is categorized into six groups according to pathogenicity mechanisms and clinical symptoms, which consist of enteropathogenic *E. coli*, enterotoxigenic *E. coli*, enteroinvasive *E. coli*, enterohemorrhagic *E. coli* (EHEC), enteroaggregative *E. coli*, and diffusely adherent *E. coli* (DEAC) [1]. *Escherichia coli* O157:H7 is a serotype of *E. coli* that expresses O antigen 157 and H antigen 7. The toxin associated with EHEC can damage the kidneys and affect the central nervous system. This toxin has been called a verocytotoxin (VT) (affecting the Vero kidney cells of the green monkey) or a Shiga toxin (similar to a toxin produced by Shigella). The toxin-producing *E. coli* is called VT-producing *E. coli* (VTEC) or Shiga toxin-producing *E. coli* (STEC) [2]. Other virulence factors of *E. coli* O157:H7 are an attaching and effacing protein (adhesin/intimin), hemolysin, catalase–peroxidase enzymes, serine protease, and type II and type III secretion systems. *Escherichia coli* O157:H7, unlike other *E. coli*, is incapable of fermenting sorbitol, lacks glucuronidase, and cannot grow at 44–45°C [3]. *Escherichia coli* O157:H7 is a foodborne and waterborne pathogen causing disease in humans upon consumption of contaminated and raw food, including raw leafy green vegetables, raw milk, or undercooked ground beef. It is not pathogenic in animals, but animals can be a reservoir of this bacterium. These pathogens are found in cattle, sheep, pigs, deer, dogs, and poultry and are released from feces. Therefore, another mode of transmitting this pathogen to humans is direct contact with carrier animal feces or consumption of contaminated products with feces during slaughterhouse processing [4, 5]. The main reservoirs of this organism are cattle and other ruminants. *Escherichia coli* O157:H7 infection in humans is a serious public health problem worldwide, especially in North America and Africa [6, 7]. The clinical manifestations associated with this bacterium are asymptomatic, non-bloody diarrhea, hemorrhagic colitis, and hemolytic uremic syndrome. Children and the elderly are a major risk group for infection and the associated complications of hemolytic uremic syndrome and death [8].

In Southern Thailand, the fighting bulls, riding horses, and fighting cocks are kept for pleasure, sport, and tourism. In addition, these pets are economic animals that support the family income in this area due to high demand in the niche market [9, 10]. These animals are well taken care of by their owners, such as daily bathing and exercise. Thus, there is very close contact between an animal and its owner. In addition, animal feces are also used as fertilizer in agriculture. Because *E. coli* O157:H7 infection can also spread from animal to person, it is serious if sports animals are infected with this bacterium. Pet owners are prone to infection with bacteria through contact with animal feces or by eating fresh vegetables contaminated with animal feces.

At present, the development of antibiotic-resistant pathogenic bacteria is mainly attributed to antibiotic use in humans and animals, including the spread of antibiotic-resistant bacteria between humans and those animals. In addition, the inappropriate or excessive use of antibiotics for treating animal diseases, disease prevention, growth promotion, and feed efficiency in livestock have accelerated the emergence of antibiotic-resistant bacteria, which can then be transmitted to humans through the food chain. Recent studies have reported an increase in the antimicrobial resistance patterns of *E. coli* O157:H7 [11–13]. However, there are no published reports on identifying *E. coli* O157:H7 in sports animals in Thailand.

As a result, this study aimed to isolate *E. coli* O157:H7 from the rectal swabs of sports animals, including fighting bulls, riding horses, and fighting cocks in Southern Thailand. In addition, antibiotic susceptibility patterns of isolated bacteria were determined. This research is particularly useful in the surveillance and prevention of this bacterial infection in sports animals and humans.

## Materials and Methods

### Ethical approval

The animal study protocol was approved by the Institutional Animal Care and Use Committee of Walailak University, Nakhon Si Thammarat, Thailand (Approval ID: WU-AICUC-63-023; approved period: Dec 14, 2020, to Dec 13, 2023).

### Study period and location

The study was conducted from January to May 2021 in Nakhon Si Thammarat province, southern Thailand.

### Sample collection

The rectal swabs were collected from sports animals (34 fighting bulls, 32 riding horses, and 31 fighting cocks). The sterile swab was inserted 1–2 inches into the rectum and rotated. The swab was transported in semisolid Cary-Blair transport media (Oxoid, Thermo Scientific™, Massachusetts, USA) to preserve the survival of enteric bacteria.

### Bacteria cultivation

The swab was cultured in MacConkey (MAC) agar (Oxoid) and incubated at 37°C in ambient air for 24 h. Suspected *E. coli* were grown in selective–differentiated MAC agar with a pink color appearance (lactose-fermenting phenotype).

### Bacterial identification and antimicrobial susceptibility testing by automation

The lactose fermenter colonies were inoculated in trypticase soy broth (TSB) and incubated for 4–6 h. TSB bacterial suspension was diluted in 0.45% sterile saline, and the density was adjusted to 0.50–0.63 MacFarland Standard using DensiCHEK™ Plus (bioMérieux, Marcy-l’Étoile, France). Bacterial suspension was identified using VITEK^®^ 2 GN card, and antimicrobial susceptibility was tested by VITEK^®^ 2 AST N194 in VITEK^®^ 2 Compact automation (bioMérieux). Bacterial identification results were interpreted using software version 5.04 (bioMérieux). Antimicrobial susceptibility profiling is reported as a minimal inhibitory concentration (MIC) value of antimicrobial agents. Clinical and Laboratory Standards Institute 2021 criteria [14] were used to interpret the antimicrobial susceptibility results in software version 5.04. *E. coli* ATCC25922 was used as a control in all experiments.

### Determination of specific characteristics of *E. coli* O157:H7

Bacteria were streaked on sorbitol MAC (SMAC) agar (Oxiod), selective and differential for *E. coli* O157:H7. After incubation at 37°C in ambient air for 24 h, *E. coli* O157:H7 grew but did not ferment sorbitol (straw-colored) [3]. *Escherichia coli* ATCC^®^ 25922 was used as a control grown with pink color caused by sorbitol fermentation efficacy. The ability of *E. coli* to grow at 44°C was determined by a streak on MAC agar and incubated for 24 h. However, *E. coli* O157:H7 does not grow well at that temperature [3]. In addition, anti-serum against H7 antigen (BD Difco™, Massachusetts, USA) was used to confirm *E. coli* O157:H7 type.

### DNA extraction

Bacterial DNA was extracted by GenElute™ Bacterial Genomic DNA Kit (Sigma-Aldrich, USA). Bacteria were cultured in 1.5 mL of TSB at 37°C for 24 h and transferred to a 2 mL tube, which is available in the kit. Next, the extraction procedure was performed according to the user manual of the commercial kit. After extraction, 200 mL of elution solution was added and incubated for 5 min at room temperature. After that, the DNA was collected by centrifugation.

### Polymerase chain reaction

The virulence genes *eaeA* (*E. coli* attaching and effacing), along with *stx1* and *stx2* (Shiga-like cytotoxins), were determined for STEC characterization. The primer sequences and polymerase chain reaction condition are shown in Table-1 [15].

**Table-1:** Polymerase chain reaction primers and conditions [15].

Genes (amplification size)	Primers (5’- 3’)	PCR condition (30 cycles)
*eaeA* (454 bp)	Forward: AAACAGGTGAAACTGTTGCC Reverse: CTCTGCAGATTAACCTCTGC	Denaturation : 94°C, 30 s Annealing : 50°C, 30 s Extention: 72°C, 60 s
*stx1* (349 bp)	Forward: CAACACTGGATGATCTCAG	Denaturation : 94°C, 30 s Annealing : 53°C, 60 s Extention: 72°C, 60 s
	Reverse: CCCCCTCAACTGCTAATA
*stx2* (110 bp)	Forward: ATCAGTCGTCACTCACTGGT	
	Reverse: CTGCTGTCACAGTGACAAA

PCR=Polymerase chain reaction

### Cell-free supernatant preparation

The *E. coli* colonies were grown in TSB for 48 h at 37°C in a shaking incubator. The bacteria culture supernatant was centrifuged at 1500× *g* at 4°C for 30 min. The supernatant was filtered through a 0.2 μm membrane filter to remove the remaining bacteria and debris [16]. Cell-free supernatant was stored at 4°C until required.

### Vero cytotoxicity test

The Vero cell line (African green monkey kidney cell; ATCC^®^ CCL81) was used to determine the cytotoxicity of toxins produced by *E. coli*. The Vero cells were cultured in Dulbecco’s modified Eagle medium (DMEM; GIBCO, USA), supplemented with 10% heat-inactivated fetal bovine serum (FBS), 100 IU penicillin/mL, and 100 g of streptomycin/mL in 25 cm^2^ culture flask at 37°C with 5% CO_2_ humidified incubator. Around 80%–90% of confluent cells were disaggregated by 1× trypsin–ethylenediaminetetraacetic acid and seeded at 1 × 10^5^ cells/well (100 μL) into 96-well plates. The plates were cultured at 37°C, 24 h, with 5% CO_2_ humidified incubator. Next, 100 μL of cell-free supernatant was added to each well and incubated for 48 h. Cell morphology was observed under an inverted microscope to check the cytotoxic effect every day [16]. Cell viability was determined by colorimetric 3-(4,5-dimethyl-2-thiazolyl)-2,5-diphenyl-2-H-tetrazolium bromide (MTT) assay [17]. The supernatant was removed by gently pipetting. After that, 10 μL of 0.5 mg/mL MTT was added and incubated for 2 h. The formazan crystal was dissolved by adding 200 μL of DMSO (dimethyl sulfoxide) and incubated for 1 h. The optical density (OD) was determined at 570 nm by using a microplate reader. The percentage of cell viability was calculated using the following formula.

Viability (%) = 100 × OD570_Sample_/OD570_Control_

## Results

### *Escherichia coli* isolation from sports animals

According to the 97 rectal swab samples (34 fighting bulls, 32 riding horses, and 31 fighting cocks), which were cultured on MAC agar, the suspected colonies of *E. coli* showed pink (lactose fermenter) with a dry and donut-shaped colony surrounded by a dark pink area of precipitated bile salts. They were selected for bacterial identification by Vitek 2^®^ Compact automation. Fifty-five samples (56.70%) were identified as *E. coli* with a percentage probability of 85% (acceptable-to-excellent confidence level). They were obtained from 19 fighting bulls (55.88%), 13 riding horses (40.63%), and 23 fighting cocks (71.13%). However, one sample (Bull H9/1) from a fighting bull had an equal confidence level (50%) for *E. coli* and *E. coli* O157, while one sample (Cock H1/4) from a fighting cock had an equal confidence level (50%) for *Salmonella enterica* subspp. diarizonae. The results are summarized in Tables-2 and 3.

**Table-2:** *Escherichia coli* from sport animal identified by Vitek 2^®^ Compact.

Sample Sources (*E. coli*/Total samples)	*E. coli* isolates by different probability			

	Excellent (96–100%)	Very good (93–95%)	Good – Accept (85–92%)	Low discrimination^a^(50%)
Fighting bulls (20/34)	3	15	1	1
Riding horses (13/32)	4	4	5	0
Fighting cocks (24/31)	5	14	4	1

a Sum of choices=100%, 2 taxa exhibit the same biopattern. Separate by supplemental testing. *E. coli=Escherichia coli*

**Table-3:** The biochemical profiles of *E. coli* O157:H7 Bull H9/1 compared to *E. coli* from other samples.

Biochemical substrates	Abbreviation	Biochemical profiles of *E. coli*			

		*E. coli* O157:H7 Bull H9/1	From other sources with different % probability		

			Excellent (96–100%)	Very good (93–95%)	Good to Acceptable (85–92%)
Ala-Phe-Pro-Arylamidase	APPA	-	- (100%)	- (100%)	- (100%)
Adonitol	ADO	-	- (100%)	-(96.97%)	- (70%)
L-Pyrrolydonyl-Arylamidase	PyrA	-	- (100%)	- (100%)	- (100%)
L-Arabitol	lARL	-	- (100%)	- (100%)	- (100%)
D-Cellobiose	dCEL	-	- (100%)	- (100%)	- (100%)
β-Galactosidase	BGAL	+	+ (100%)	+(100%)	+(100%)
H_2_ S production	H2S	-	- (100%)	- (100%)	- (100%)
β-N-Acetyl-Glucosaminidase	BNAG	-	- (100%)	- (100%)	- (100%)
Glutamyl Arylamidase pNA	AGLTp	-	- (100%)	- (100%)	- (100%)
D-Glucose	dGLU	+	+ (100%)	- (100%)	+ (100%)
γ-Glutamyl-Transferase	GGT	-	- (91.67%)	- (72.72%)	+ (100%)
Fermentation/Glucose	OFF	+	+ (100%)	+ (100%)	+ (90%)
β-Glucosidase	BGLU	-	- (100%)	- (100%)	+ (100%)
D-Maltose	dMAL	+	+ (91.67%)	+ (100%)	- (100%)
D-Mannitol	dMAN	+	+ (100%)	+ (100%)	+ (100%)
D-Mannose	dMNE	+	+ (100%)	+ (100%)	+ (100%)
β-Xylosidase	BXYL	-	- (100%)	- (100%)	+ (100%)
β-Alanine arylamidase pNA	BAlap	-	- (100%)	- (100%)	- (100%)
L-Proline Arylamidase	ProA	+	- (83.33%)	- (81.82%)	- (100%)
Lipase	LIP	-	- (100%)	- (100%)	+ (70%)
Palatinose	PLE	-	- (100%)	- (100%)	- (100%)
Tyrosine Arylamidase	TyrA	+	+ (91.67%)	+ (90.91%)	+ (100%)
Urease	URE	+	+ (100%)	+ (100%)	+ (100%)
D-Sorbitol	dSOR	-	+ (100%)	+ (100%)	+ (90%)
Saccharose/Sucrose	SAC	+	- (100%)	+ (90.91%)	+ (50%)
D-Tagatose	dTAG	-	- (100%)	- (93.94%)	- (100%)
D-Trehalose	dTRE	+	+ (91.67%)	+ (100%)	+ (100%)
Citrate (sodium)	CIT	-	- (100%)	- (96.97%)	+ (100%)
Malonate	MNT	-	- (100%)	- (100%)	- (80%)
5-Keto-D-Gluconate	5KG	-	- (100%)	- (72.72%)	- (80%)
L-Lactate alkalinization	lLATk	+	+ (100%)	+ (100%)	+ (100%)
α-Glucosidase	AGLU	-	- (100%)	- (100%)	- (100%)
Succinate alkalinization	SUCT	+	+ (100%)	+ (100%)	+ (100%)
β-N-Acetyl-Galactosaminidase	NAGA	-	- (100%)	- (100%)	- (100%)
α-Galactosidase	AGAL	+	+ (100%)	+ (100%)	+ (100%)
Phosphatase	PHOS	+	+ (66.67%)	- (84.85%)	+ (80%)
Glycine Arylamidase	GlyA	+	+ (100%)	+ (96.97%)	+ (100%)
Ornithine Decarboxylase	ODC	+	+ (66.67%)	+ (63.63%)	+ (50%)
Lysine Decarboxylase	LDC	+	+ (83.33%)	+ (93/94%)	+ (100%)
L-Histidine assimilation	lHISa	+	- (100%)	- (100%)	- (100%)
Courmarate	CMT	+	+ (100%)	+ (100%)	+ (100%)
β-Glucoronidase	BGUR	+	+ (100%)	+ (100%)	+ (100%)
O/129 Resistance (comp.vibrio.)	O129R	+	+ (83.33%)	+ (66.67%)	+ (90%)
Glu-Gly-Arg-Arylamidase	GGAA	-	- (100%)	- (100%)	- (100%)
L-Malate assimilation	lMLTa	+	- (83.33%)	- (90.91%)	+ (80%)
Ellman	ELLM	+	+ (100%)	+ (100%)	+ (100%)
L-Lactate assimilation	lLATa	+	- (100%)	+ (96.97%)	- (70%)

*E. coli=Escherichia coli*

### *Escherichia coli* O157:H7 biopattern and confirmation test

To confirm the bacterial species of Bull H9/1, the biochemical profile was analyzed by using Vitek^®^ 2 Compact, as shown in Table-3. Four substrates, namely, D-sorbitol (dSOR), urease, L-lactate assimilation (lLATa), and β-glucuronidase (BGUR), were used to differentiate between *E. coli* and *E. coli* O157. *Escherichia coli* O157 is dSOR-negative and lLATa-positive, in contrast to *E. coli*. However, the conventional phenotypic test, growth on SMAC, was used to confirm *E. coli* O157. The colony of Bull H9/1 on SMAC agar was straw-colored, while a pink colony was observed on MAC. Moreover, it did not grow well at 44°C and reacted with anti-serum to H7 antigen. *Escherichia coli* O157 cannot produce BGUR or is BGUR-negative. However, a previous study reported that *E. coli* O157:H7, which was isolated from animals and humans, was BGUR-positive [18]. Moreover, all *E. coli* isolates were urease-positive in this study.

### *Escherichia coli* O157:H7 virulence genes’ identification

The representative virulence genes, *eaeA*, *stx1*, and *stx2*, of *E. coli* O157:H7 were determined in this study. The PCR results of Bull H9/1 isolate were positive with *eaeA* and *stx2* genes. This indicated that this strain is STEC or VTEC. Therefore, to confirm this toxin’s activity on the Vero cell line, the Vero cytotoxicity test was conducted.

### Vero cytotoxicity of cell-free supernatant from *E. coli* O157:H7

Cell-free supernatant of *E. coli* Bull H9/1 demonstrated significant toxic effects on the Vero cell compared to control cells. The cytopathic effects of cell-free-supernatant-treated Vero cells were round shriveled cells within 48 h and showed complete syncytial degeneration within 72 h.

**Figure-1:**
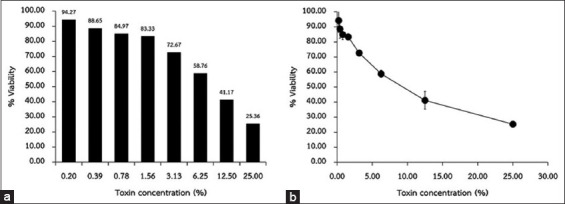
Vero cytotoxicity test of various concentrations of cell-free supernatant *E. coli* O157:H7 Percentage of cell viability was determined by MTT assay (a) bar graph and (b) line graph.

The cytotoxicity test of various concentrations of cell-free supernatant of *E. coli* Bull H9/1 by two-fold serial dilution from 25% to 0.2% is depicted in Figure-1. The cell-free supernatant’s CC_50_ (50% cytotoxicity concentration) on Vero cells was 9.5% of toxin concentration.

### Antimicrobial susceptibility patterns

**Figure-2:**
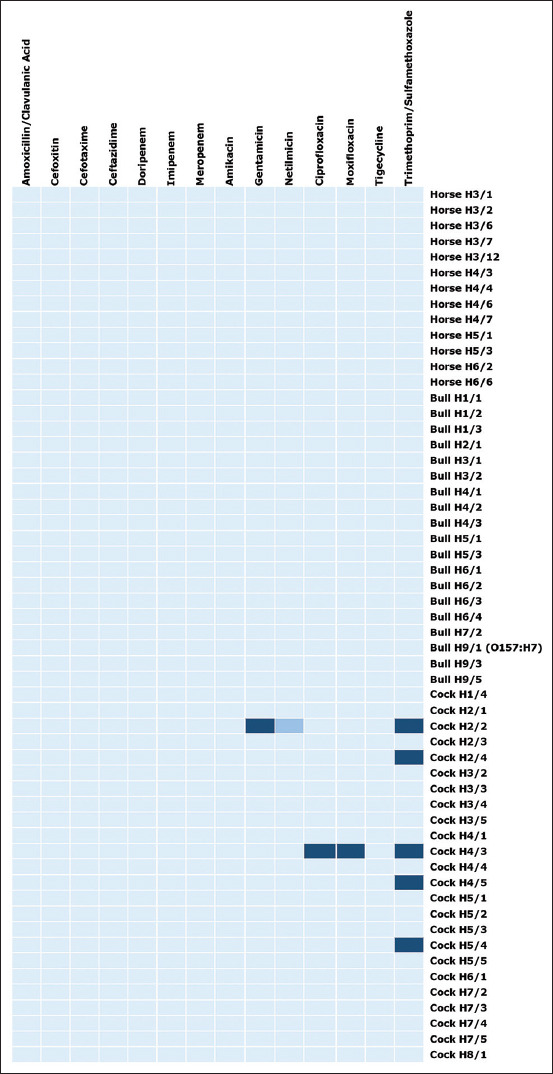
Heat map of antimicrobial susceptibility patterns of 55 isolates of *Escherichia coli* and one isolate of *E. coli* O157:H7 from sport animals. Each row represented each isolate.

Antimicrobial susceptibility patterns of *E. coli* isolated from sports animals were determined by Vitek 2^®^ Compact automation and reported as MICs. The results revealed that five out of 55 (9.09%) *E. coli* isolates were resistant to antimicrobial agents (Figure-2). Interestingly, all five isolates (21.74%) were collected from fighting cocks. The antimicrobial susceptibility pattern’s heat map was also generated for both 55 isolates of *E. coli* and one isolate of *E. coli* O157:H7 (Figure-2). *Escherichia coli* Cock H4/3 is only one of five (20%) isolates resistant to three antimicrobial agents (ciprofloxacin, moxifloxacin, and trimethoprim/sulfamethoxazole). However, ciprofloxacin and moxifloxacin belong to the same antimicrobial class. Therefore, this strain is not multidrug-resistant (MDR) bacteria. *Escherichia coli* Cock H2/2 shows resistance to two antimicrobial agents (gentamicin and trimethoprim/sulfamethoxazole) and intermediate resistance to netilmicin. The other three isolates (Cock H2/4, H4/5, and 5/4) are resistant to trimethoprim/sulfamethoxazole.

The MICs of gentamicin, ciprofloxacin, and moxifloxacin against antimicrobial agent-resistant *E. coli* were ≥16, ≥4, and ≥8 μg/mL, respectively, whereas the MICs of trimethoprim/sulfamethoxazole of all isolates were ≥320 μg/mL. In addition, the MIC of intermediate resistance to netilmicin is 16 μg/mL (Table-4). When the MIC values were considered, most of them were equal to or lower than the lowest concentration of antimicrobial agent tested levels. Moreover, the extended-spectrum beta-lactamase (ESBL) production was also determined. However, no isolate of *E. coli* produced ESBL in this study.

**Table 4:** Susceptibility patterns of *E. coli* showed as MIC values which were determined by Vitek 2^®^ Compact automation.

Antibiotics	Probability (no.)	Minimal inhibition concentration (µg/mL)																			ESBL	
	
		0.1	0.3	0.5	1	2	4	8	16	≤12	≤0.25	≤0.5	≤1	≤2	≤4	≤20	≥320	≥16	≥8	≥4	Pos	Neg
Amoxicillin/Clavulanic acid	Excellent (12)						5	1						6							
	Very Good (33)						8	3						22							
	Good-Acceptable (10)						2	1						7								
Cefoxitin	Excellent (12)														12							
	Very Good (33)							1							32							
	Good-Acceptable (10)												10									
Cefotaxime	Excellent (12)												12									
	Very Good (33)												33									
	Good-Acceptable (10)																					
Ceftazidime	Excellent (12)												12									
	Very Good (33)												33									
	Good -Acceptable (10)												10									
Doripenem	Excellent (12)									12												
	Very Good (33)									33												
	Good-Acceptable (10)									10												
Imipenem	Excellent (12)										12											
	Very Good (33)										33											
	Good-Acceptable (10)										10											
Meropenem	Excellent (12)										12											
	Very Good (33)										32											
	Good-Acceptable (10)			1																		
Amikacin	Excellent (12)													12								
	Very Good (33)						1							32								
	Good-Acceptable (10)													10								
Gentamicin	Excellent (12)												11					**1**				
	Very Good (33)			1							32											
	Good-Acceptable (10)																					
Netilmicin	Excellent (12)					1			1				10									
	Very Good (33)					6							27									
	Good-Acceptable (10)												10									
Ciprofloxacin	Excellent (12)										11									**1**		
	Very Good (33)			2							31											
	Good-Acceptable (10)				1						9											
Moxifloxacin	Excellent (12)										11								**1**			
	Very Good (33)			2		5					26											
	Good-Acceptable (10)			2		2					6											
Tigecycline	Excellent (12)											12										
	Very Good (33)											33										
	Good-Acceptable (10)											10										
Trimethoprim/Sulfamethoxazole	Excellent (12)															10	**2**					
	Very Good (33)															31	**2**					
	Good-Acceptable (10)															9	**1**					
ESBL production	Excellent (12)																					12
	Very Good (33)																					33
	Good-Acceptable (10)																					10

Underline letters represented the mode of MIC values; Red-letter represented intermediate susceptible; Red with bold letters represented resistance. MIC=Minimun inhibitory concentration, *E. coli*=Escherichia coli

## Discussion

Sports animals, including fighting bulls, riding horses, and fighting cocks, are economically and closely related to humans. Animal-fighting sports are popular in a number of countries, such as India, Mexico, Spain, and Japan [19–22], including Thailand [9, 10]. The sports animal caregivers have a high risk of microbial transmission due to close contact with the sporting animal [23]. Therefore, the surveillance study for possible pathogens’ reservoirs in those animals is beneficial. A previous study reported that *E. coli* O157:H7 in cattle feces and contact surfaces in the butcher shop was 2.8% [24]. This is in agreement with our study, which found *E. coli* O157:H7 in one fighting bull by rectal swab culture from 34 rectal swabs of fighting bulls (2.94%). However, *E. coli* O157:H7 was not found in riding horses and fighting cocks. It is not surprising as the main reservoir of *E. coli* O157:H7 reported previously was cattle [25]. However, there have been reports that *E. coli* O157:H7 has been isolated from goats, sheep, pigs, chickens, horses, and other animals [26–30]. Moreover, it persists and grows in farm environments, including straw, soil, and water [31].

Biochemical substrates utility profile-based VITEK^®^ 2 Compact automation was used in this study. It is a reliable method and has been used in several previous veterinary studies [32–34]. However, Crawford-Miksza *et al*. [35] reported the misidentification of the variant biotype of *E. coli* O157:H7 as *Escherichia fergusonii*. Our study found a low discrimination of *E. coli* O157:H7 with a 50% possibility. Therefore, confirming the characteristic of *E. coli* O157 by classical microbiological diagnostic is essential. The ability to ferment dSOR, growth at 44°C, and the presence of H7 antigen were found in our study. Moreover, our study gave details of the biochemical profile of *E. coli* O157:H7 compared to *E. coli* with different percentage probability (Table-3).

The *E. coli* O157:H7 is considered EHEC when it can produce Vero toxins or Shiga toxins (Stx). The name of toxins is related to their cytopathic effect on Vero cells or relatedness of the toxin of *Shigella dysenteriae* [2]. The outbreak of Shiga toxin-producing *E. coli* O157:H7 has been reported worldwide [6, 7]. Stx toxins are classified into two main groups: Stx1 and Stx2. These two groups are further divided into nine subgroups, namely, Stx1, Stx1c, Stx1d, Stx2, Stx2c, Stx2d, Stx2d_activatable_, Stx2e, and Stx2f [2]. Tahamtan *et al*. [36] reported the prevalence and distribution of *stx* genes in STEC in cattle in Iran. They found that both genes were detected in 34.93% of STEC isolates, while a single gene of stx1 or stx2 was found in 10.27% and 53.42% of STEC isolates, respectively. Moreover, the *stx* genes are found predominantly in animals during the warm season. In this study, *E. coli* O157:H7 Bull H9/1 was found positive with the *stx2* gene. Moreover, it was also positive, meaning that the *eaeA* gene encoding adhesin intimin influences the establishment of bacterial adherence to the intestinal epithelial cells [37]. In Southern Thailand, *E. coli* O157 was reported to be carrying *stx* and *eae* at high frequencies in beef sold in markets [38, 39]. Interestingly, previous data indicated that Stx2-producing *E. coli* O157 isolates cause more severe effects on infected persons than Stx1-producing isolates [40]. Purified Stx2 is thousands of times more toxic to renal epithelial cells than Stx1 [41]. Our study not only detected the presence of genotype but also presented the cytopathic effects on Vero cells by the dose-dependent response (Figure-1). The result can confirm the expression of Stx2 toxin, which has high toxicity on Vero cells (renal epithelial cells).

As we understand, the use of antimicrobial agents for therapeutic and especially non-therapeutic purposes for farm animals will promote the incidence of antimicrobial agent resistance through selective pressure. The majority of previous research was conducted on farm animals whose sole purpose was to produce food [42–44]. Antimicrobial agents used in animal farms for food production are not intended to promote growth; however, if infectious diseases are not treated promptly, they can negatively impact the productivity and economy of affected businesses. Sports animals are frequently provided with advanced care, and antimicrobial agent resistance can have a negative social and economic impact on the owners. Our study analyzed and showed the antimicrobial susceptibility patterns of *E. coli* and *E. coli* O157:H7, as summarized in Table-4 and the heat map in Figure-2. Antimicrobial agent-resistant *E. coli* strains were found in only fighting cocks. One of five strains was resistant to three types of antimicrobial agents. However, it cannot be categorized as MDR bacteria as gentamicin and netilmicin are aminoglycoside drugs, and as per its definition, a multidrug-resistant bacterium is resistant to three or more antimicrobial classes [45].

However, the antimicrobial agent used in our study was limited. If more drugs are used, the chances of finding drug-resistant bacteria will increase. Interestingly, all five strains of *E. coli* were resistant to trimethoprim/sulfamethoxazole. The previous studies reported trimethoprim/sulfamethoxazole-resistant *E. coli* isolates from broiler chickens in many countries such as Canada, Bangladesh, and Spain [46–48], including Thailand [48]. Mooljuntee *et al*. [49] reported that 26.7% of trimethoprim/sulfamethoxazole-resistant *E. coli* were found in broiler chickens, while our study reported 21.74%. To the best of our knowledge, our study is the first to report antimicrobial agent-resistant *E. coli* from fighting cocks. This finding indicated that drug use in fighting cock for treatment or prevention purposes must be considered. Interestingly, the prevalence of gentamicin-resistant *E. coli* was one from 55 total strains (1.82%) and 24 fighting cock isolated strains (4.17%). It was less than that isolated from humans (20%), as Wu *et al*. [50] described. However, they proposed the distribution of gentamicin-resistant *E. coli* from animals to humans because gentamicin resistance genes are on the transferable plasmid [51].

## Conclusion

This is the first study that revealed the identification of *E. coli* O157:H7, which is an EHEC and produces Vero toxins (Shiga toxin) and intimin from the fighting bull. Moreover, antimicrobial agent-resistant *E. coli* from the rectal swab of fighting cocks in Southern Thailand were determined. Because the fighting bulls and fighting cocks are very close to humans, the STEC O157:H7 or antimicrobial agent-resistant *E. coli* might be transmitted to humans by direct or indirect contact. Therefore, it is essential to prevent the dissemination of *E. coli* O157:H7 or antimicrobial agent-resistant *E. coli* in sports animals and humans. Moreover, the antimicrobial agent resistance of *E. coli* indicates that the drug administration in fighting cock for treatment or prevention must be considered.

## Data Availability

The supplementary data can be available from the corresponding author on a reasonable request.

## Authors’ Contributions

WKK and TW: Collected samples. WKK, WM, SW, KS, SC, and NN: Cultured bacteria and microbial sensitivity. WKK and JS: Molecular analysis and cell culture. WKK and TW: Designed the experiment. WKK and TW: Received the grant and managed the project. WKK and JS: Drafted the manuscript. All authors have read and approved the final manuscript.
